# Risk factors associated with preterm birth among singletons following assisted reproductive technology in Australia 2007–2009–a population-based retrospective study

**DOI:** 10.1186/s12884-014-0406-y

**Published:** 2014-12-07

**Authors:** Xu K Xu, Yueping A Wang, Zhuoyang Li, Kei Lui, Elizabeth A Sullivan

**Affiliations:** School of Medical Sciences, University of New South Wales, Kensington Campus, Sydney, NSW 2052 Australia; School of Women’s and Children’s Health, University of New South Wales, Randwick Hospitals Campus, Sydney, NSW 2031 Australia; Faculty of Health, University of Technology Sydney, City Campus, Sydney, NSW 2007 Australia; Department of Newborn Care, Royal Hospital for Women, Sydney, NSW 2031 Australia

**Keywords:** Assisted reproductive technology, Singleton, Preterm birth, In vitro fertilisation, Risk factor

## Abstract

**Background:**

Preterm birth, a leading cause of neonatal death, is more common in multiple births and thus there has being an increasing call for reducing multiple births in ART. However, few studies have compared risk factors for preterm births amongst ART and non-ART singleton birth mothers.

**Methods:**

A population-based study of 393,450 mothers, including 12,105 (3.1%) ART mothers, with singleton gestations born between 2007 and 2009 in 5 of the 8 jurisdictions in Australia. Univariable and multivariable logistic regression models were conducted to evaluate socio-demographic, medical and pregnancy factors associated with preterm births in contrasting ART and non-ART mothers.

**Results:**

Ten percent of singleton births to ART mothers were preterm compared to 6.8% for non-ART mothers (P < 0.01). Compared with non-ART mothers, ART mothers were older (mean 34.0 vs 29.7 yr respectively), less socio-economically disadvantaged (12.4% in the lowest quintile vs 20.7%), less likely to be smokers (3.8% vs 19.4%), more likely to be first time mothers (primiparous 62.4% vs 40.5%), had more preexisting hypertension and complications during pregnancy. Irrespective of the mode of conception, preexisting medical and pregnancy complications of hypertension, diabetes and antepartum hemorrhages were consistently associated with preterm birth. In contrast, socio-demographic variables, namely young and old maternal age (<25 and >34), socioeconomic disadvantage (most disadvantaged quintile Odds Ratio (OR) 0.95, 95% Confidence Interval (CI): 0.77–1.17), smoking (OR 1.12, 95%CI: 0.79–1.61) and priminarity (OR 1.19, 95% CI: 1.05–1.35, AOR not significant) shown to be associated with elevated risk of preterm birth for non-ART mothers were not demonstrated for ART mothers, even after adjusting for potential confounders. Nonetheless, in multivariable analysis, the association between ART and the elevated risk for singleton preterm birth persisted after controlling for all included confounding medical, pregnancy and socio-economic factors (AOR 1.51, 95% CI: 1.42–1.61).

**Conclusions:**

Preterm birth rate is approximately one-and-a-half-fold higher in ART mothers than non-ART mothers albeit for singleton births after controlling for confounding factors. However, ART mothers were less subject to the adverse influence from socio-demographic factors than non-ART mothers. This has implications for counselling prospective parents.

## Background

Preterm birth (gestation of <37 weeks) is a global public health problem [1,2]. More than one-third of neonatal deaths (death in the first four weeks of life) are directly attributable to preterm birth in 2010 [2,3]. Survivors of preterm birth are at increased risk of neonatal morbidity [4,5] and long-term adverse health outcomes including cerebral palsy, cognitive impairment [6], neurosensory deficits [3] and pulmonary disease [3]. Further, the cost to the health sector of preterm infants is substantial [7], and the hospital cost increases exponentially with decreasing gestational age [8].

Assisted reproductive technology (ART), defined by the World Health Organization and the International Committee Monitoring Assisted Reproductive Technologies as treatment that involves the handling of human oocytes (eggs) and sperms or embryos in a laboratory to establish pregnancies [9], has increasingly been used to treat infertility. It was estimated that ART has contributed to the birth of over 5 million liveborn babies worldwide [10]. ART has been associated with a significantly higher rate of multiple gestational pregnancies which are associated with increased risk of preterm birth [11]. However, elevated risk of preterm birth following ART has also been found for singleton pregnancies compared with non-ART singleton pregnancies [12-14].

International studies have shown that the socio-demographic characteristics, pregnancy-related complications and medical conditions of women giving birth after ART differ from non-ART mothers [15,16]. However, few studies have investigated whether preterm birth, a multifactorial condition [17], is associated with varied sets of risk factors in these two groups.

Australia has one of the highest rates of ART utilisation as well as the practice of single embryo transfer in the world [18-20]. In 2011, 61,158 ART treatment cycles were undertaken in Australia, representing 12.9 cycles per 1,000 women of reproductive age (15–44 years) [21]. National data shows that over 17% of ART babies were preterm, which was markedly higher than the proportion of preterm babies (8.3%) born in Australia in 2010 [22,23]. It is therefore timely to examine the association of ART and preterm birth.

Since 2007, the National Perinatal Data Collection (NPDC) which collects information on all births in Australia, has included information on use of ART [24]. The present study is the first large population-based study to investigate the association between ART and the risk of singleton preterm birth. The aims of this study were to: (1) Compare the socio-demographic, medical and pregnancy characteristics of ART and non-ART mothers; (2) Investigate the association between ART and preterm birth; and (3) Identify and compare risk factors associated with preterm birth for ART and non-ART mothers.

## Methods

### Data source

Perinatal data were provided on an annual basis by the health authorities in all Australian states and territories and compiled into the NPDC [24-26]. The NPDC includes national information on the pregnancy and childbirth of all Australian mothers, and the characteristics and outcomes of their babies. Namely NPDC includes maternal demographic factors; mothers’ medical conditions prior to pregnancy; complications during pregnancy; ART status; labour, mode of birth and perinatal outcomes. A birth is defined as a stillbirth or live birth of at least 20 weeks of gestation or at least 400 grams birthweight.

### Data quality

All singleton births during the study period between 1 January 2007 to 31 December 2009 from 5 of the 8 states and territories where data on ART use was available (Victoria, Queensland, Western Australia, Tasmania and Australian Capital Territory) were extracted from NPDC. Of these, 19,118 records that did not state ART status (4.6%) and 59 records that did not state gestational age (<0.1%). The data quality statement for the NPDC was published in reports *Australia’s mothers and babies 2007, 2008 and 2009* [24-26].

Data about most variables was with minor missings. However, maternal pre-pregnancy body mass index (BMI) and smoking during pregnancy were not required data items in the NPDC, so BMI was available for women in only one jurisdiction between 2008 and 2009 (N = 119,257), accounting for 30.31% of all women in the study population. Whether a woman smoked during pregnancy was indicated for women in two jurisdictions between 2007 and 2009, and one jurisdiction between 2008 and 2009 (N = 253,444), accounting for 64.42% of all women in the study population. Maternal pre-existing medical conditions including essential hypertension, diabetes mellitus and epilepsy were available in all but one jurisdiction in 2009 (N = 387,256), accounting for 98.43% of the study population.

### Study population

The inclusion criteria for the study population consisted of singleton births during the study period from the 5 jurisdictions. The 19,118 records that did not state ART status and 59 records that did not state gestational age were excluded.

The final study population consisted of 393,450 women who gave birth to singleton babies, accounting for 45.6% of all singleton births in Australia during the three-year period.

### Variables

The main outcome variable was preterm birth, this dichotomous variable was defined as < 37 completed weeks of gestation. The other outcome variable is gestational age, a continues variable.

The exposure variable was a dichotomous variable of ART status (yes or no). Other risk factors were selected based on published literature and were classified into three conceptual groups; (1) Demographic factors including maternal age, country of birth (Australia or overseas); smoking status during pregnancy (yes, no and not stated); pre-pregnancy BMI; remoteness (major cities, inner regional, outer regional, remote, very remote or other); health insurance type (public, private cover or other), Socio-Economic Indexes for Areas (SEIFA) (categorised into quintiles) [27]; and parity (grouped as primiparous and multiparous); (2) Maternal pre-existing medical conditions including essential hypertension, diabetes mellitus and epilepsy; and (3) Complications arising in pregnancy including hypertensive disorders in pregnancy, gestational diabetes and antepartum haemorrhage. The socio-economic status was measured by Socio-economic Indexes for Areas (SEIFA). The Index of Relative Socio-Economic Disadvantage (IRSD). The indexes ranked geographic areas across Australia in terms of their socio-economic characteristics. The SEIFA indexes were created by combining information collected in the five-yearly Census of Population and Housing in Australia [26].

### Statistical analysis

Descriptive statistics were generated to compare maternal characteristics and outcomes between ART and non-ART mothers; student t-test was used for continuous variables; and Pearson’s χ^2^-test was used for categorical variables.

The odds of singleton preterm births in ART and non-ART mothers were compared using univariable and multivariable logistic regression models. The multiple logistic regression model was adjusted for maternal age, country of birth, smoking status during pregnancy, SEIFA, parity, health insurance type, remoteness, essential hypertension, diabetes mellitus, epilepsy, hypertensive disorders in pregnancy, gestational diabetes and antepartum haemorrhage. Such analysis was conducted within the total study population and within populations stratified by women’s parity.

Univariable and stepwise multivariable logistic regression analyses were conducted separately in the ART and non-ART mothers to calculate crude and adjusted odds ratios for each risk factor in relation to preterm birth.

Missing and not applicable data were included in regression models as a separate group. Items with less than 20 cases in any category were excluded from the model. Data analysis was performed using IBM Statistical Package for Social Sciences (SPSS), version 20. Findings with a P-value <0.05 or a CI not including 1 were considered statistically significant.

### Ethics approval

Ethics approval was received for the study from the Human Research Ethics Committee of the University of New South Wales (HREC Ref: # HC11024) and Australian Institute of Health and Welfare (HREC Ref: # EC2011/1/5).

## Results

### Characteristics of women who gave birth to singletons by ART status

Of the 393,450 women who had singleton births, 12,105 (3.1%) gave birth following ART treatment, and 381,345 (96.9%) did not use ART.

The socio-demographic characteristics of mothers who underwent ART treatment were significantly different to those of mothers who did not have ART treatment (Table 1). The average age of ART mothers was significantly higher than for non-ART mothers (34.0 years versus 29.7 years, P < 0.01), and ART mothers were more likely to be aged ≥35 years (47.1% versus 21.4%, P < 0.01) at the time of delivery. ART mothers were more likely to be first-time mothers (62.4%) compared with non-ART mothers (40.5%; P < 0.01). Further, compared with non-ART mothers, higher proportions of ART mothers were born in Australia, were non smokers, had a higher socioeconomic status (SES), were non-obese, resided in major cities and had private health insurance (Table 1). However, of the three pre-existing medical conditions examined, only essential hypertension was significantly more prevalent among ART mothers (1.2% versus 0.9%, P = 0.01) (Table 2). The obstetric characteristics differed according to mothers’ mode of conception. The prevalence of hypertensive disorders in pregnancy, gestational diabetes and antepartum haemorrhage were significantly higher among ART mothers than non-ART mothers (6.1% versus 4.4% P < 0.01, 7.5% versus 5.0% P < 0.01, and 5.0% versus 2.8% P < 0.01 respectively) (Table 2).

**Table 1:** **Sociodemographic characteristics of singleton birth mothers by ART status, Australia, 2007–2009**

	**Non-ART**		**ART**		**P**
	**(N = 381,345)**		**(N = 12,105)**		
	**No.**	**%**	**No.**	**%**	
**Maternal age (years)**					
Mean ± SD	29.7 ± 5.7		34.0 ± 4.8		<0.01
Less than 24	76,182	20.0	267	2.2	<0.01
25–34	223,494	58.6	6,131	50.7	
35–44	81,216	21.3	5,535	45.7	
45 and over	439	0.1	172	1.4	
**Maternal country of birth**					
Australia	294,196	77.6	9,671	80.2	<0.01
Overseas	85,083	22.4	2,389	19.8	
**BMI (kg/m** ^**2**^ **)** ^**(a)**^					
Less than 18.5	4,749	4.3	163	3.9	<0.01
18.5–24.9	49,913	45.6	2,079	49.7	
25.0–29.9	30,819	28.2	1,086	26.0	
30.0–34.9	14,336	13.1	492	11.8	
35.0–39.9	6,199	5.7	223	5.3	
40.0 and over	3,470	3.2	140	3.4	
**SEIFA Index of Disadvantage**					
1 (Most disadvantaged)	78,473	20.7	1,498	12.4	<0.01
2	62,969	16.6	1,528	12.7	
3	83,153	22.0	2,620	21.7	
4	87,116	23.0	3,093	25.7	
5 (Least disadvantaged)	66,987	17.7	3,320	27.5	
**Remoteness**					
Major cities	244,632	64.2	8,778	72.6	<0.01
Inner regional	80,205	21.1	2,113	17.5	
Outer regional	44,867	11.8	1,009	8.3	
Remote	7,082	1.9	155	1.3	
Very remote	4,252	1.1	41	0.3	
Other^(b)^	307		9		
**Smoking status during pregnancy** ^**(c)**^					
Yes	47,179	19.4	321	3.8	<0.01
No	196,661	80.7	8,181	96.2	
**Parity**					
Primiparous	154,519	40.5	7,549	62.4	<0.01
Multiparous	226,826	59.5	4,556	37.6	
**Health insurance**					
Public	256,511	67.6	3,274	27.1	<0.01
Private	122,987	32.4	8,812	72.9	
Other ^(d)^	1,847		19		

(a) Maternal pre-pregnancy body mass index (BMI) was available in only one jurisdiction between 2008 and 2009 (N = 119,257), accounting for 30.3% of the study population.

(b) Includes 289 non-Australian residents and 27 not stated cases.

(c) Smoking during pregnancy was indicated in two jurisdictions between 2007 and 2009, and one jurisdiction between 2008 and 2009 (N = 253,444), accounting for 64.42% of the study population.

(d) Includes 1,124 not applicable and 742 not stated cases.

**Table 2:** **Medical and obstetric characteristics of singleton birth mothers by ART status, Australia, 2007–2009**

	**Non-ART**		**ART**		**P**
	**(N = 381,345)**		**(N = 12,105)**		
	**No.**	**%**	**No.**	**%**	
**Essential hypertension** ^(a)^					
Yes	3,468	0.9	137	1.2	0.01
No	371,934	99.1	11,717	98.8	
**Diabetes mellitus** ^**(a)**^					
Yes	2,447	0.7	78	0.7	0.93
No	372,954	99.4	11,776	99.3	
**Epilepsy** ^**(a)**^					
Yes	2,007	0.5	54	0.5	0.24
No	373,395	99.5	11,800	99.5	
**Hypertensive disorders in pregnancy**					
Yes	16,850	4.4	742	6.1	<0.01
No	364,495	95.6	11,363	93.9	
**Gestational diabetes**					
Yes	19,075	5.0	904	7.5	<0.01
No	362,270	95.0	11,201	92.5	
**Antepartum haemorrhage**					
Yes	10,699	2.8	600	5.0	<0.01
No	370,632	97.2	11,505	95.0	

(a) Maternal pre-existing medical conditions including essential hypertension, diabetes mellitus and epilepsy were available in all but one jurisdiction in 2009 (N = 387,256), accounting for 98.43% of the study population.

The proportion of preterm births in ART mothers (10.1%) was significantly higher compared with non-ART mothers (6.8%) (P < 0.01); and the average length of gestation was shorter in ART pregnancies compared with non-ART pregnancies (38.4 weeks versus 38.9 weeks, P < 0.01) (Table 3).

**Table 3:** **Perinatal outcomes of singleton births by ART status, Australia, 2007–2009**

	**Non-ART**		**ART**		**P**	**Total**	
	**(N = 381,345)**		**(N = 12,105)**			**(N = 393,450)**	
	**No.**	**%**	**No.**	**%**		**No.**	**%**
**Birth status**							
Live birth	378,408	99.2	12,009	99.2	0.78	390,417	99.2
Stillbirth	2,937	0.8	96	0.8		3,033	0.8
**Gestational age (weeks)**							
Mean ± SD	38.9 ± 2.2		38.4 ± 2.5		<0.01	38.9 ± 2.2	
<37	25,871	6.8	1,219	10.1	<0.01	27,090	6.9
≥37	355,474	93.2	10,886	89.9		366,360	93.1
**Gender**							
Female	184,576	48.4	5,923	48.9	0.26	190,499	48.4
Male	196,675	51.6	6,181	51.1		202,856	51.6

### Association between ART and preterm birth

Table 4 shows a positive association between ART treatment and preterm birth (OR 1.54; 95% CI: 1.45–1.63); the risk of preterm birth was only marginally reduced after multiple potential confounders were adjusted (AOR 1.51; 95% CI: 1.42–1.61). Sub-group analysis on data from the jurisdiction where BMI was available indicates the odds of preterm birth for ART mothers were 59% higher than for non-ART mothers (AOR 1.59; 95% CI: 1.42–1.77).

**Table 4:** **Association between ART status and preterm birth among singleton birth mothers, Australia, 2007–2009**

**ART status**	**All singleton births**	**Preterm births (N = 27,090)**				
	**(N = 393,450)**	**N (%)**	**OR (95% CI)**	**AOR** ^**(a)**^ **(95% CI)**	**AOR** ^**(b)**^ **(95% CI)**	**AOR** ^**(c)**^ **(95% CI)**
Yes	12,105	1,219 (10.1)	1.54 (1.45–1.63)	1.56 (1.46–1.66)	1.62 (1.52–1.73)	1.51 (1.42–1.61)
No	381,345	25,871 (6.8)	1	1	1	1

(a) Multiple logistic regression model adjusted for maternal age.

(b) Multiple logistic regression model adjusted for maternal age, country of birth, smoking status during pregnancy, SEIFA, parity, health insurance type and remoteness.

(c) Multiple logistic regression model adjusted for maternal age, country of birth, smoking status during pregnancy, SEIFA, parity, health insurance type, remoteness, essential hypertension, diabetes mellitus, epilepsy, hypertensive disorders in pregnancy, gestational diabetes and antepartum haemorrhage.

Compared with non-ART singletons, ART singletons were at 44% (AOR 1.44, 95% CI: 1.33–1.56) and 60% (AOR 1.60, 95% CI: 1.43–1.78) of increased odds of being preterm among primiparous and multiparous mothers respectively.

### Risk factors for preterm birth by ART status

The differences in characteristics of ART and non-ART mothers suggest there may be variations in risk factors for preterm birth between the two groups. Therefore, risk factors relating to preterm birth were analysed separately among non-ART and ART mothers (Tables 5 and 6).

**Table 5:** **Demographic, medical and obstetric characteristics and preterm births among non-ART singleton birth mothers, Australia, 2007–2009**

**Characteristics**	**All singleton births (N = 381,345)**	**Preterm births**			
		**No.**	**%**	**OR (95% CI)**	**AOR** ^**(a)**^ **(95% CI)**
**Maternal age**					
Less than 25	76,182	6,012	7.9	1.27 (1.23–1.31)	1.12 (1.08–1.16)
25-34	223,494	14,130	6.3	1	1
35-44	81,216	5,673	7.0	1.11 (1.08–1.15)	1.13 (1.09–1.17)
45 and over	439	44	10.0	1.65 (1.21–2.25)	1.49 (1.08–2.06)
**Smoking status during pregnancy**					
Smoked	47,179	4,569	9.7	1.64 (1.59–1.70)	1.55 (1.50–1.61)
Did not smoke	196,661	12,050	6.1	1	1
**Maternal country of birth**					
Overseas	85,083	5,464	6.4	0.93 (0.90–0.96)	NS
Australia	294,196	20,211	6.9	1	
**SEIFA Index of Disadvantage**					
1 (Most disadvantaged)	78,473	5,919	7.5	1.28 (1.23–1.34)	1.14 (1.09–1.19)
2	62,969	4,565	7.3	1.23 (1.18–1.28)	1.11 (1.06–1.17)
3	83,153	5,543	6.7	1.12 (1.08–1.17)	1.05 (1.00–1.10)
4	87,116	5,516	6.3	1.06 (1.02–1.11)	1.02 (0.98–1.07)
5 (Least disadvantaged)	66,987	4,007	6.0	1	1
**Parity**					
Primiparous	154,519	11,397	7.4	1.17 (1.14–1.20)	1.13 (1.10–1.17)
Multiparous	226,826	14,474	6.4	1	1
**Remoteness**					
Major cities	244,632	15,951	6.5	1	1
Inner regional	80,205	5,732	7.2	1.10 (1.07–1.14)	1.00 (0.97–1.04)
Outer regional	44,867	3,216	7.2	1.11 (1.06–1.15)	1.01 (0.96–1.05)
Remote	7,082	528	7.5	1.15 (1.06–1.26)	1.04 (0.95–1.14)
Very remote	4,252	371	8.7	1.37 (1.23–1.53)	1.13 (1.01–1.27)
**Health insurance**					
Public	256,511	18,612	7.3	1	1
Private	122,987	7,207	5.9	0.80 (0.77–0.82)	0.89 (0.86–0.92)
**Essential hypertension**					
Yes	3,468	511	14.7	2.41 (2.19–2.65)	2.05 (1.86–2.27)
No	371,934	24,921	6.7	1	1
**Diabetes mellitus**					
Yes	2,447	606	24.8	4.62 (4.21–5.06)	4.13 (3.75–4.56)
No	372,954	24,826	6.7	1	1
**Epilepsy**					
Yes	2,007	219	10.9	1.10 (1.00–1.21)	1.49 (1.29–1.73)
No	373,395	25,213	6.8	1	1
**Hypertensive disorders in pregnancy**					
Yes	16,850	2,778	16.5	2.92 (2.80–3.05)	2.94 (2.81–3.08)
No	364,495	23,093	6.3	1	1
**Gestational diabetes**					
Yes	19,075	1,639	8.6	1.31 (1.24–1.38)	1.27 (1.20–1.34)
No	362,270	24,232	6.7	1	1
**Antepartum haemorrhage**					
Yes	10,699	3,311	31.0	6.92 (6.62–7.22)	7.11 (6.80–7.43)
No	370,632	22,557	6.1	1	1

(a) Stepwise multiple logistic regression model adjusted for maternal age, country of birth, smoking status during pregnancy, SEIFA, parity, health insurance type, remoteness, essential hypertension, diabetes mellitus, epilepsy, hypertensive disorders in pregnancy, gestational diabetes and antepartum haemorrhage.

NS: statistically not significant.

**Table 6:** **Demographic, medical and obstetric characteristics and preterm births among ART singleton birth mothers, Australia, 2007–2009**

**Characteristics**	**All singleton births (N = 12,105)**	**Preterm births**			
		**No.**	**%**	**OR (95% CI)**	**AOR** ^**(a)**^ **(95% CI)**
**Maternal age**					
Less than 25	267	23	8.6	0.86 (0.56–1.33)	NS
25-34	6,131	604	9.9	1	
35-44	5,535	566	10.2	1.04 (0.92–1.18)	
45 and over	172	26	15.1	1.63 (1.06–2.49)	
**Smoking status during pregnancy**					
Smoked	321	35	10.9	1.12 (0.79–1.61)	NS
Did not smoke	8,181	803	9.8	1	
**Maternal country of birth**					
Australia	9,671	941	9.7	1	NS
Overseas	2,389	268	11.2	1.17 (1.02–1.35)	
**SEIFA Index of Disadvantage**					
1 (Most disadvantaged)	1,498	140	9.4	0.95 (0.77–1.17)	NS
2	1,528	169	11.1	1.15 (0.94–1.40)	
3	2,620	282	10.8	1.12 (0.94–1.32)	
4	3,093	297	9.6	0.98 (0.83–1.16)	
5 (Least disadvantaged)	3,320	324	9.8	1	
**Parity**					
Primiparous	4,556	414	9.1	1.19 (1.05–1.35)	NS
Multiparous	7,549	805	10.7	1	
**Remoteness**					
Major cities	8,778	910	10.4	1	^(b)^
Inner regional	2,113	191	9.0	0.86 (0.73–1.01)	
Outer regional	1,009	98	9.7	0.93 (0.75–1.16)	
Remote	155	14	9.0	0.86 (0.49–1.49)	
Very remote	41	2	4.9	0.44 (0.11–1.84)	
**Health insurance**					
Public	3,274	367	11.2	1	NS
Private	8,812	847	9.6	0.84 (0.74–0.96)	
**Essential hypertension**					
Yes	137	27	19.7	2.21 (1.45–3.39)	2.16 (1.39–3.36)
No	11,717	1,169	10.0	1	1
**Diabetes mellitus**					
Yes	78	21	26.9	3.32 (2.01–5.50)	2.74 (1.61–4.67)
No	11,776	1,175	10.0	1	1
**Epilepsy**					
Yes	54	9	16.7	1.79 (0.87–3.67)	^(b)^
No	11,800	1,187	10.1	1	
**Hypertensive disorders in pregnancy**					
Yes	742	175	23.6	3.05 (2.55–3.66)	3.21 (2.67–3.87)
No	11,363	1,044	9.2	1	1
**Gestational diabetes**					
Yes	904	125	13.8	1.48 (1.22–1.81)	1.43 (1.17–1.76)
No	11,201	1,094	9.8	1	1
**Antepartum haemorrhage**					
Yes	600	189	31.5	4.68 (3.89–5.62)	4.83 (4.01–5.82)
No	11,505	1,030	9.0	1	1

(a) Stepwise multiple logistic regression model adjusted for maternal age, country of birth, smoking status during pregnancy, SEIFA, parity, health insurance type, remoteness, essential hypertension, diabetes mellitus, epilepsy, hypertensive disorders in pregnancy, gestational diabetes and antepartum haemorrhage.

(b) Item removed from the model due to less than 20 cases in any category.

NS: statistically not significant.

All of the factors examined, except for the mother’s country of birth, were associated with preterm births among non-ART mothers (Table 5). The most pronounced demographic factor associated with preterm birth following non-ART treatment was smoking during pregnancy (AOR 1.55, 95% CI: 1.50–1.61) (Table 5).

However, sociodemographic factors, including maternal age, parity, smoking during pregnancy, socioeconomic status, remoteness and patients’ insurance type, previously seen to play a role in predisposing preterm birth among non-ART mothers were no longer associated with preterm birth among ART mothers (Table 6).

ART and non-ART mothers share some common risk factors for preterm birth: the pre-existing maternal medical conditions and complications arising during pregnancy, including pre-existing hypertension (AOR 2.16 (1.39–3.36) and 2.05 (1.86–2.27)), diabetes mellitus (2.74 (1.61–4.67) and 4.13 (3.75–4.56)), hypertensive disorders in pregnancy (3.21 (2.67–3.87) and 2.94 (2.81–3.08)), gestational diabetes (1.43 (1.17–1.76) and 1.27 (1.20–1.34)) and antepartum haemorrhage (4.83 (4.01–5.82) and 7.11 (6.80-7.43)) (Tables 5 and 6).

Antepartum haemorrhage showed the strongest association with preterm birth in both ART (AOR 4.83, 95%CI: 4.01–5.82) and non-ART (AOR 7.11, 95% CI: 6.80–7.43) mothers, although the association was stronger in the non-ART group (Tables 5 and 6). Diabetes mellitus and hypertensive disorders in pregnancy were identified as the second strongest risk factors among ART and non-ART mothers respectively (Tables 5 and 6).

## Discussion

As Australia was an early adopter of single embryo transfer in clinical practise, it is timely to investigate whether ART is independently associated with preterm birth and whether risk factors related to preterm birth vary by the mode of conception. There were three main findings of this study: (1) The characteristics of women who gave birth to singleton babies in Australia varied by the mode of conception; (2) The odds of singleton preterm birth among women who gave birth after ART in Australia was approximately one-and-a-half-fold compared with non-ART mothers; and (3) Risk factors associated with preterm birth differ between ART and non-ART mothers.

Findings from this study suggest that ART is independently associated with singleton preterm birth. Notwithstanding the inability to control for the number of embryos transferred, the odds of preterm birth in women with singleton birth following ART treatment is 1.51-fold (95% CI: 1.42–1.61) compared to non-ART mothers. This finding was consistent with the 1.54-fold in a recent systematic review (95% CI: 1.47–1.62) [28]; but was comparatively lower than the approximately two-fold risk of preterm birth found in three earlier systematic reviews published in 2004 [12-14]. The reduction in risk may be partially explained by increased use of single embryo transfer resulting in fewer preterm births among singletons as shown by our group [29-31].

The characteristics of women giving birth after ART in Australia were markedly different to their non-ART counterparts, this finding is consistent with two earlier international studies from the US [15,16], ART mothers were older, more socioeconomically privileged and more likely to be first-time mothers; the proportion of essential hypertension and complications in pregnancy were also higher in this group. The differences in characteristics between the two groups suggest that risk factors associated with preterm birth in the ART group may differ from those for non-ART mothers. An array of socio-demographic factors was found to be associated with preterm birth among non-ART mothers. Interestingly, the important demographic factors predisposing preterm birth among non-ART mothers, namely age, socioeconomic status, smoking and parity were no longer found to be associated with preterm birth among ART mothers. This may be due to the relative homogeneity of the ART group. This result is consistent with Tepper’s study which is, to our knowledge, the only previous study that investigated risk factors associated with singleton preterm birth by the use of ART. The US study also found that socioeconomic factors had weaker associations with preterm birth among the ART mothers than among non-ART mothers [15].

In addition to gestational hypertension and diabetes in pregnancy identified by Tepper and colleagues [15], the present study found that essential hypertension, diabetes mellitus and antepartum haemorrhage were, independently associated with increased odds of preterm birth regardless of the mode of conception. Prevention and proper management of these conditions before and during pregnancy for both ART and non-ART mothers are important.

The current study revealed socioeconomic inequity related to the use of ART in Australia. Although Australia is one of the few countries in the world that have ART services meeting the level of demand [32], the utilisation of these services varied according to one’s socioeconomic background. More than two-thirds of ART mothers were covered by private health insurance, while the majority of the non-ART group were public patients. There also is a positive association between elevated risk of preterm birth and low SES among non-ART mothers. This finding is comparable with international studies that used maternal educational attainment and neighbourhood income as indicators of SES [33-35]. The current study demonstrated that mothers from the lowest SES quintile had a 14% excess risk of preterm birth compared with mothers from the highest quintile (AOR 1.14, 95% CI: 1.09–1.19). This result is very similar to the finding in a Canadian study (AOR 1.14, 95% CI: 1.10–1.17) [35]. A low SES is associated with unintended/unwanted pregnancies, which were shown to play a role in increasing the risk of preterm birth [36,37].

The absence of socio-demographic and economic risk factors among mothers who gave birth after ART may be related to their overall high SES. In a Massachusetts study, Schieve and colleagues [16] demonstrated that ART mothers tended to receive more adequate antenatal care initiated from an earlier stage of pregnancy compared with non-ART mothers. Adequate antenatal care was shown to be protective against preterm birth [38-40]. A Finnish study also attributed the improved perinatal outcomes after IVF treatment over time to the more intense care received by ART mothers [41]. Furthermore, unintended pregnancies do not play a role in predisposing preterm birth in ART mothers because all pregnancies following ART are planned*.* The more adequate antenatal care received by ART mothers may at least in part compensate for the adverse effects resulting from giving birth at an advanced age and being primiparous.

Similar to the age distribution of ART mothers in Massachusetts [15], the current study found that 47.1% of ART mothers were over 35 years old; in comparison, approximately 21.4% of non-ART mothers were over 35 years old. Further, as also found in Tepper and colleagues’ study, the association between maternal age and preterm birth was present among non-ART mothers but was absent among ART mothers. However, the absence of such association should be interpreted with caution as advancing maternal age remains an important risk factor associated with declining success rate of clinical pregnancy after an IVF procedure [42]. Additionally, women aged more than 35 are more likely to have double embryo transfer [43], a practice leading to the high incidence of multiple gestation which can result in vanishing twin and singletons that are preterm [44]. Finally, other unfavourable obstetric complications, such as chromosomal abnormality, increase markedly with advancing maternal age [45]. Therefore, women should not postpone their motherhoods albeit that age is not a significant risk factor for preterm birth after ART treatment.

Compared with non-ART mothers, the proportion of ART mothers who smoked during pregnancy was significantly less (3.8% versus 19.4%, P < 0.01). Infertile couples seeking ART treatment tend to experience higher level of anxiety upon losing the pregnancy [46], it is therefore understandable that ART women were more mindful of factors such as cigarette smoking that predisposes miscarriage [47]. As shown by Tepper and colleagues, the percentage of smoking decreased from 5.6% prior conception to 1.7% after conception among ART women, while this decrement was milder among non-ART women (from 17.0% to 9.8%) [15].

As anticipated, smoking was found to be a risk factor predisposing preterm births among mothers who did not use ART. Surprisingly, the current study did not demonstrate an association between smoking and preterm birth among mothers who gave birth after ART treatment. A possible type II error may be caused by the small number of preterm birth among ART mothers who smoke (35 in total). Given that there is a dose–response relationship between the quantity of cigarettes smoked and preterm birth [48], if ART mothers who smoke were exposed to smaller quantities of cigarettes compared with non-ART mothers, the statistical model might fail to detect the effect of smoking on preterm birth among ART mothers. Notwithstanding, couples should be counselled to cease smoking during pregnancy to minimise risk to the unborn fetus.

The strength of the current study is that it is a population-based study containing validated, singleton birth records for five out of eight Australian jurisdictions. Up to 0.4 million mothers were included in the study population, accounting for approximately 45% of all singleton births in the three-year period. The age of mothers and the prevalence of preterm birth among singleton infants following ART treatment included in the study resume the entire Australian and New Zealand*.* The average age of women at the time of delivery was 34.0 ± 4.8 in this study, comparable with 34.8 in 2007 [49] and 35.0 in 2008 and 2009 [20,50] based on data from *The Australian and New Zealand Assisted Reproduction Database*. The 10.1% preterm birth prevalence in the current study is also similar to the levels in Australia and New Zealand, where the prevalence of singleton preterm birth following ART treatment was 10.5% in 2007 [49], 10.7% in 2008 [50] and 9.9% in 2009 [49]. Therefore the results can be generalised to the nation with the unique socio-demographic and economic context of each State and Territory taken into consideration, especially in Northern Territory that has the highest percentage of Indigenous women who give birth and second highest smoking rate [24-26]. With more than twelve thousand ART mothers included in the study, the diminished associations between multiple sociodemographic factors and preterm birth were unlikely to be due to reduced population size.

We acknowledge some limitations of the current study. Data were not available on the number of embryos transferred. Data for smoking during pregnancy and BMI were missing in some states and territories during the study period. Nevertheless, the quality of data from jurisdictions that provided BMI and smoking information were good, accounting for approximately 30% and 64% of the study population respectively. Sub-group analysis on data where BMI was available showed similar result to the AOR produced for the whole study population.

Although caesarean section is an obstetric precursor for preterm birth [17], this association was not investigated in this study. A caesarean section could result from emergency or various clinical complications, however in the data collection it was not possible to identify if an elective caesarean section was indeed planed or not and its indications. Future studies could examine this link.

The current study did not include information on previous obstetric history, such as whether the pregnancy was subsequent to a prior preterm birth, which has been shown in the literature to be independently associated with preterm birth [51,52]. A South Australian study restricted the study population to women in their first singleton pregnancy so as to remove the confounding effect of multiple pregnancies, parity and previous preterm birth [53]. In the current study, adjustment was made for parity in the regression model instead of stratifying for primiparous women. Sub-group analysis showed a comparable association between preterm birth and ART for both primiparous and multiparous women.

As a cross sectional data collection, the NPDC dataset does not provide information regarding to separate deliveries for a same woman. Future studies using longitudinal data would consider linking deliveries for the same women.

From a clinical perspective, couples in Australia who are considering using ART should be informed about the potential of increased risk of preterm birth even for singleton births. Antenatal care guidelines should include consideration of a woman’s mode of conception reflecting the variation in risk factors for ART and non-ART mothers, and the excess risk of preterm birth for ART mothers.

The findings of this study underscore the possibility that the mechanism causing preterm birth following ART treatment is different from preterm birth in the general population even after elimination the confounding factor of multiple gestation. The literature has postulated that the underlying causes of infertility may explain the elevated risk of preterm births among ART mothers [54,55]. Further research is required. In addition, further studies may focus on determining whether there are variations in risk factors for other adverse birth outcomes, such as very preterm birth (<32 weeks of gestation), by the use of ART.

## Conclusions

The current study identified ART as an independent risk factor for preterm birth in an Australian setting and that there are variations of risk factors associated with preterm birth for ART and non-ART mothers. Couples should be informed about the elevated risk of preterm birth associated with ART, and criteria for ART mothers should be developed that identify individuals who are at high risk of preterm birth. Further studies may focus on assessing the associations between the stated risk factors and other adverse perinatal outcomes in ART mothers.

## Abbreviations

AIHW: Australian Institute of Health and Welfare
AOR: Adjusted odds ratio
ART: Assisted reproductive technology
BMI: Body mass index
CI: Confidence interval
IVF: In vitro fertilisation
NPDC: National perinatal data collection
OR: Odds ratio
SEIFA: Socio-economic indexes for areas
SES: Socioeconomic status
SPSS: Statistical package for social sciences
